# Large-scale extraction of brain connectivity from the neuroscientific literature

**DOI:** 10.1093/bioinformatics/btv025

**Published:** 2015-01-20

**Authors:** Renaud Richardet, Jean-Cédric Chappelier, Martin Telefont, Sean Hill

**Affiliations:** ^1^Blue Brain Project, Brain Mind Institute and ^2^School of Computer and Communication Sciences, Ecole Polytechnique Fédérale de Lausanne (EPFL), Lausanne, Switzerland

## Abstract

**Motivation:** In neuroscience, as in many other scientific domains, the primary form of knowledge dissemination is through published articles. One challenge for modern neuroinformatics is finding methods to make the knowledge from the tremendous backlog of publications accessible for search, analysis and the integration of such data into computational models. A key example of this is metascale brain connectivity, where results are not reported in a normalized repository. Instead, these experimental results are published in natural language, scattered among individual scientific publications. This lack of normalization and centralization hinders the large-scale integration of brain connectivity results. In this article, we present text-mining models to extract and aggregate brain connectivity results from 13.2 million PubMed abstracts and 630 216 full-text publications related to neuroscience. The brain regions are identified with three different named entity recognizers (NERs) and then normalized against two atlases: the Allen Brain Atlas (ABA) and the atlas from the Brain Architecture Management System (BAMS). We then use three different extractors to assess inter-region connectivity.

**Results:** NERs and connectivity extractors are evaluated against a manually annotated corpus. The complete *in litero* extraction models are also evaluated against *in vivo* connectivity data from ABA with an estimated precision of 78%. The resulting database contains over 4 million brain region mentions and over 100 000 (ABA) and 122 000 (BAMS) potential brain region connections. This database drastically accelerates connectivity literature review, by providing a centralized repository of connectivity data to neuroscientists.

**Availability and implementation:** The resulting models are publicly available at github.com/BlueBrain/bluima.

**Contact:** renaud.richardet@epfl.ch

**Supplementary information:** Supplementary data are available at *Bioinformatics* online.

## 1 Introduction

Accessing the vast amounts of data and knowledge embedded in the previous decades of neuroscience publications is essential for modern neuroinformatics. Making these data and knowledge accessible can help scientists maintain a state-of-the-field perspective and improve efficiency of the neuroscientific process by reducing repeated experiments and identifying priorities for new experiments. Efforts to build models of neural circuitry must integrate such data into the model building process to benefit from the data of many years of prior research. In the case of metascale brain region connectivity, thousands of experiments have been published in scientific journals. However, these have not been systematically normalized and registered in a central repository of brain region connectivity. Thus, researchers resort to manual searches on PubMed that are very time consuming.

### 1.1 Brain connectivity data integration

Brain connectivity data consist of information about one brain region projecting nerve fibers to another region and forming synaptic connections. Additional metadata includes, for example connection strength, animal species and experimental methods.

Brain connectivity data can be integrated from different sources. For the mouse brain, one central source is the *Allen Mouse Brain Connectivity Atlas* [AMBCA, Oh *et al.* (2014)]. As of today, the Allen Institute has published 1772 standardized connectivity experiments tracking axonal projections in the adult mouse brain by two-photon imaging of fluorescently labeled neurons. Experimental results have been normalized to a coordinate-based reference space and are freely available to researchers via a publicly accessible API (connectivity.brain-map.org). The AMBCA is a very valuable source of connectivity data because of the consistency of the experimental methods, the standardized brain region naming, the availability of the data and the overall high level of quality of the data.

A second central source of connectivity data comes from curated databases of the published literature. For the rat brain, the most important is the *Brain Architecture Management System* [BAMS, Bota and Swanson (2008)]. Neuroscientists from the BAMS project have manually curated over 600 scientific articles. They analyzed each article (including tables, images and supplementary materials) and assessed the quality of the experiment. Finally, they normalized brain region mentions to the BAMS ontology and recorded the connectivity data into a database (including directionality and strength).

One other major source of connectivity data is the analysis of neuroscientific articles. This is commonly performed by *manual search* on databases like PubMed or Google Scholar. The search, curation and integration of these articles might be a manageable task for a researcher focused on one or a few brain regions, but it does not scale for whole-brain models. Furthermore, manual search for brain region connections has several disadvantages. First, the naming of brain regions is *diverse*, making it difficult to search for brain region names. These nomenclatures rely on different detection methods (e.g. Nissl staining, immunostaining, functional magnetic resonance imaging and diffusion tensor imaging) that result in different sizes and shapes of brain regions.

Another disadvantage of manual search is its low recall (*Recall* is the ratio of the number of relevant records retrieved to the total number of relevant records.). It is likely to miss a significant part of the brain regions because it *lacks synonym expansion*. For example, exact search for ‘Basolateral amygdala nucleus’ (17 results on PubMed) will neither return results from the synonym ‘Basolateral nucleus of the amygdala’ (297 results) nor from the Latin name ‘Nucleus amygdalae basolateralis’ (8 results). Another reason for low recall is the *lack of abbreviation expansion*. For example, when searching for ‘Ventral tegmental area’, the abbreviated form ‘VTA’ will not be retrieved. A random sample corpus of 179 full-text articles from the *Journal of Comparative Neurology* contained on average 91.6 brain regions mentions and 29.7 abbreviations of brain regions per article. This represents a maximal possible 32% increase in recall when performing abbreviation expansion (Note, however, that an article containing an abbreviated brain region might still be returned by a manual search, since abbreviations are almost always explicitly defined in an article, so the expected increase is smaller.). Additionally, for a significant number of articles in PubMed, *only the abstract* is indexed and searchable, not the full article body. On the aforementioned corpus, the abstracts contained on average 2.8 brain region mentions. This represents a possible 32-fold increase in recall when using full-text instead of abstracts.

In terms of precision (*Precision* is the fraction of retrieved records that are relevant.), a manual search will return all brain regions that co-occur within the same document. Most of these *co-occurrences* do not necessarily represent true neurophysiological connections but simply that two brain regions are mentioned in the same document. At the abstract level, French *et al.* (2012) found that only 2.2% of the co-occurrences represent true connections. At the sentence level, the proportion raises to only 13.3%. Thus, the precision of manual search is expected to be quite low, meaning that researchers will waste time in manually post-processing the search result and probably discard most retrieved co-occurrences.

### 1.2 Information Extraction

**Fig. 1.**
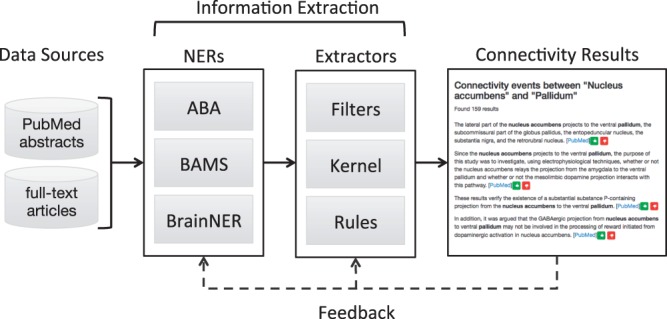
Overview of datasets, methods and models. Three named entity recognizers (NER) identify and normalize brain region mentions: *BAMS* and *ABA* (lexical-based) and *BraiNER* (machine learning-based). Three different extractors predict the connectivity probability of brain region co-occurrences: *Filters* takes a top–down filtering approach, *Kernel* is a machine learning-based classifier and *Rules* consists of hand-written extraction rules. Connectivity results are presented in a searchable web interface. In the future, feedback from the interface can be used to retrain the NERs and extractors for continuous model improvement

One way to improve manual literature search is to make use of automated information extraction (IE). IE aims at extracting structured information from unstructured text. It facilitates the manual search of brain connectivity data by analyzing very large numbers of scientific articles and proposing to the neuroscientist a list of brain regions potentially connected. In the present case, the IE process is divided in two phases: named entity recognition (NER) and relation extraction (Fig. 1).

To build a brain region NER, the first and simplest approach is to match entities from a list of brain regions (lexical-based NER). There exist a plethora of brain region ontologies and taxonomies that can be used as lexica (see Section 2.1). However, most of these have been designed to *structure and organize* brain regions but not to serve as a resource for IE. Typically, they lack appropriate synonyms and can be too specific, resulting in low recall [e.g. ‘Entorhinal area, lateral part, layer 6a’ is a brain region from the Allen Brain Atlas (ABA) ontology that is very unlikely to be found in a scientific article].

A second and more sophisticated approach to building a NER is to train a machine learning model on annotated corpora providing examples of brain regions. The model relies on so-called *features* to take a decision on whether a group of words represent a brain region. Features can be, for example that a word starts with a capital letter, whether the word belongs to a neuroanatomical lexicon or whether the previous word is a verb. A model usually includes several hundreds different features and model training consists in learning which combinations of features are most likely to identify a brain region. Once a model has been trained on an annotated corpus, it can be used to identify brain regions on new, unseen text. The advantage is that the model will match complex brain region names, even if they are not present in any lexica, for example ‘contralateral prepositus hypoglossal nucleus’ or ‘distal parts of the inferior anterior cerebellar cortex’. However, a drawback of this machine learning approach is that corpus annotations, required to train the model, are very time-consuming and require domain experts.

Machine learning NERs have been successfully developed in the biomedical field for entities like proteins (Campos *et al.*, 2013), chemicals (Jessop *et al.*, 2011), species (Gerner *et al.*, 2010) and anatomical entities (Pyysalo and Ananiadou, 2014). For brain regions, NER models have been published by Burns *et al.* (2008) and French *et al.* (2012). They both rely on linear chain conditional random fields (CRFs), with model features based on morphological, lexical, syntactic and contextual information. French’s model achieves a state-of-the-art performance of 86% recall and 92% precision on a training corpus of 1377 abstracts with 18 242 brain region annotations.

Once named entities have been identified, we normalize them, so that, e.g. both ‘diencephalon’ and ‘interbrain’ resolve to the same entity. Normalization can be performed by automatically or manually attaching synonyms to lexical-based NER or by performing morpho-syntactic transformations on the brain regions extracted by a NER. For example, French and Pavlidis (2012) used transformation to remove prefixes that specify hemispheres (‘Contralateral inferior olivary’ is transformed into ‘Inferior olivary’) or to remove neuroanatomical direction specifiers (‘Caudal cuneate nucleus’ is transformed into ‘Cuneate nucleus’).

The second and last IE step involves relationship extraction. It aims at classifying co-occurrences between two brain region entities and predicting whether they represent neurophysiological connections (It is worth noting that IE only predicts whether the author *reports* a connection between two brain region, not whether the connection *actually exists*, which is out of the scope of such an IE system.). Models for relationship extraction include rule-based and supervised machine learning approaches. Relationship extraction between two biomedical entities is a current research topic, applied to problems like protein–protein interaction (Krallinger *et al.*, 2011) or pathway curation (Ohta *et al.*, 2013). The difficulty of the task resides in the complexity of the relation between two or more brain regions (Table 1). French *et al.* (2012) developed and evaluated several models to extract brain region connectivity. Their simple co-occurrence-based methods yielded high recall but low precision, whereas the advanced machine learning models recalled 70.1% of the sentence-level connectivity statements at 50.3% precision. More complex models based on dependency parsing were successfully evaluated by French *et al.* (2012) but discarded because of their high computational cost.

**Table 1.** Example of sentences exhibiting connectivity statements between brain regions

Sample sentence	Connectivity statement, comment
The nucleus accumbens (AC) receives projections from both the substantia nigra (SN) and the ventral tegmental area (VTA) (Dworkin, 1988).	(SN, VTA) → AC
Substantial numbers of tyrosine hydroxylase-immunoreactive cells in the dorsal raphe nucleus (DR) were found to project to the nucleus accumbens (AC) (Stratford and Wirtshafter, 1990).	DR → AC
The dentate gyrus (DG) is, of course, not only an input link between the entorhinal cortex (Ent) and the hippocampus proper (CAs) but also a major site of projection from the hippocampus (CA), as are the amygdala (Amg), entorhinal cortex (Ent) and septum (Spt) (Izquierdo and Medina, 1997).	CAs → DG → Ent, (CA, Amg, Ent, Spt) → DG Complex, long range relationships
This latter nucleus (N?), which projects to the striatum (CP), receives inputs from motor cortex (MO) as well as the basal ganglia (BG) and is situated to integrate these and then provide feedback to the basal ganglia (BG) (Strutz, 1987).	MO → N? → CP, BG ↔ N? Anaphora: ‘latter nucleus (N?)’ was defined in previous sentence
In this review, we summarize a classic injury model, lesioning of the perforant path, which removes the main extrahippocampal input to the dentate gyrus (Perederiy and Westbrook, 2013).	Injury model, not normal conditions
The most commonly proposed mechanism is that the periaqueductal gray of the midbrain (PAG) or the cerebral cortex (Cx) have descending influences to the spinal cord (SpC) to modulate pain transmission at the spinal cord (SpC) level (Andersen, 1986).	PAG → SpC, Cx → SpC ‘proposed’ implies an hypothesis, not a finding

Abbreviations have been manually added.

Our work builds on top of French *et al.* (2009, 2012) and French and Pavlidis (2012) and extends it in several aspects: ensemble of three different extractors and application to a large corpus of over 8 billion words.

## 2 Methods

To build a database of brain region connectivity data from the literature, two steps are required. First, NERs identify brain region mentions in text and normalize them to a standard brain region ontology. Second, extractors are developed to determine whether two brain region co-occurrence mentions are semantically connected. Finally, the connectivity results are stored in a database to be accessible by researchers.

### 2.1 Brain region NERs

Three different NERs have been developed to identify and normalize brain region mentions (Table 2). The first lexical NER (ABA) consists of all 1197 entities from the Allen Mouse Brain Atlas [Allen Reference Atlas, version 2 (2011), Mouse Brain Atlas Ontology]. Lexical matching is performed using UIMA ConceptMapper, with order dependant lookup, longest contiguous match and a stemmer that removes endings of words longer than three characters. As discussed in Section 1.1, the atlas is designed to structure and organize brain regions within the Allen Brain Institute and not as a lexical resource for IE. Thus, the ABA NER contains no synonyms. To retrieve more relevant data (and improve recall), a second NER (ABA-SYN) is automatically augmented with corresponding synonyms found in several lexica of rodent brain region: BAMS (Bota and Swanson, 2008), Hof *et al.* (2000), Neuronames (Bowden and Dubach, 2003), Paxinos and Watson (2006), Swanson (2004). For example, for the ABA entity ‘Pontine gray’, the Neuronames lexicon also contains several synonyms (e.g. ‘Nuclei pontis’) that are added back to the corresponding ABA entry. This results in a 3-fold increase in recall between ABA and ABA-SYN. To further improve recall, ABA-SYN is manually augmented with brain region mentions appearing frequently in scientific articles but not included in ABA-SYN. Additionally, abbreviation expansion is performed on the input text using a machine learning-based model [hidden Markov model, Movshovitz-Attias and Cohen (2012)]. The same procedure for ABA is applied to the BAMS ontology (Swanson, 2004).

**Table 2.** NERs for brain regions

NER name	Description	Brain regions	Terms
ABA	Lexicon from ABA Institute	1197	1197
ABA-SYN	ABA + automated synonyms enrichment from other lexica	1197	3882
BAMS	Lexicon from BAMS, version Swanson (2004)	832	832
BAMS-SYN	BAMS + automated synonyms enrichment from other lexica	832	2705
BraiNER	Machine learning-based NER (linear chain CRF)	(*∞*)	(*∞*)

The third brain region NER, BraiNER, extends the work from French *et al.* (2009) and relies on a supervised machine learning model [linear chain CRF (McCallum, 2002)]. The model is trained on WhiteText (www.chibi.ubc.ca/WhiteText), a manually annotated corpus of brain region mentions composed of 1377 PubMed abstracts from the *Journal of Comparative Neurology*, containing 18 242 brain region mentions. Inter-annotator agreement was evaluated by French *et al.* (2009) by two curators for a subset of the documents and reached 90.7% and 96.7% for strict and lenient matching, respectively.

The model features from French *et al.* (2009) are primarily derived from existing neuroanatomical lexica. These include, for example lexical features such as the presence of directionality words like *dorsal* or *ventral* or morphological features like the word length or whether it contains only lowercase letters, numbers or special characters. BraiNER uses the following additional features: the presence of species information in the document (identified using the Linnaeus NER (Gerner *et al.*, 2010)] and the presence of a measure entity [e.g. a measure like *10 mm* or *10(-7) molar*]. Indeed, a qualitative analysis of the performance of BraiNER on full-text articles revealed that measures were often incorrectly labeled as brain regions (false positives). Furthermore, several other features are developed to improve robustness on full-text articles, motivated by the large amount of false positives when analyzing full-text articles, in particular when processing bibliographical information or tables.

### 2.2 Connectivity Extractors

Connectivity extractors are binary classifiers. They take as input a sentence containing at least two brain region mentions (as identified by the above NERs) and take a decision whether the sentence enunciates a connection between these two brain regions. The models developed in this article focus on extracting connections with high precision. They are limited to brain regions that are co-located within the same sentence (no anaphora resolution) and do not extract the directionality of the connection.

Three different approaches are developed to classify connectivity statements (Fig. 1). (i) FILTER considers all possible co-occurrences of brain regions and subsequently applies filters to remove unlikely ones. More precisely, it starts with all permutations of brain regions within a sentence and then keeps only nearest neighbors, that is: only co-occurrences that are located closest to each other. After that, co-occurrences in sentences longer than 500 characters are removed, since longer sentences are unlikely to be meaningful sentences. Similarly, sentences containing more than seven brain regions are removed, since they are too complex to extract. These filters were developed based on our experience with full-text articles that can contain very long sentences or lists of brain regions. Finally, only sentences containing one of the following trigger character sequences are retained: *afferent, efferent, project, connecti, pathway* and *inputs*. (ii) KERNEL relies on a supervised classifier [shallow linguistic kernel (Giuliano *et al.*, 2006), identical to French *et al.* (2012)] that requires only shallow parsing information such as word occurrences and part-of-speech tags. (iii) RULES consists of nine rules of the kind ‘*projection from the region* *A (of the region* *B) to the region* *C and the region* *D’*. Here, the strategy is to identify characteristic sentence constructs and thus achieve a very high precision at the cost of recall. Rules are manually crafted using the Apache UIMA Ruta workbench (Kluegl *et al.*, 2014). The Ruta workbench enables a rapid and iterative development of lexical rules.

## 3 Experiments and results

We begin by quantitatively evaluating the performance of the brain region NERs and connectivity extractors against annotated corpora. We then build a database by applying these models on three different corpora. We describe the database and conclude by performing a qualitative evaluation of the database against the connectivity data from ABA.

### 3.1 NERs evaluation

All five NERs described in Table 2 are evaluated against *WhiteText* (French *et al.*, 2009) (Table 3). Two types of evaluations are performed: exact comparison (meaning that the span of a proposed brain region must exactly match a manually annotated brain region) and lenient comparison (meaning that the span of an identified brain region may be equal or smaller than a manually annotated brain region). When performing exact comparison, lexical-based NERs score low on both precision and recall. For both NERs enriched with synonyms (ABA-SYN and BAMS-SYN), recall is significantly higher (21.9% and 17.5%, respectively). Using lenient comparison, lexical-based NERs score much higher on precision (between 89.8% and 92.1%). However, recall is low, even with synonyms (between 16.2% and 34.2%). One reason why lexical-based NERs do not achieve perfect precision is that they wrongly label implicit brain regions (e.g. they will label ‘midbrain’ in ‘midbrain ventral tegmental area’ or ‘midbrain lateral tegmental field’). Another reason is that they sometimes label brain regions that are more specific (e.g. ‘brachium of the superior colliculus’ was labeled, whereas the gold-standard only includes ‘superior colliculus’).

**Table 3.** Performance comparison of brain region NER models against the WhiteText corpus (partially matching spans)

Model	Exact comparison			Lenient comparison		
	Precision	Recall	*F* score	Precision	Recall	*F* score
ABA lexicon	58.4%	11.1%	18.6%	89.9%	16.9%	28.5%
ABA-SYN lexicon	58.4%	21.9%	31.9%	**92.1%**	34.2%	49.9%
BAMS lexicon	61.1%	11.0%	18.6%	90.7%	16.2%	27.5%
BAMS-SYN lexicon	61.3%	17.5%	27.2%	89.8%	25.5%	39.7%
WhiteText (French *et al.*, 2009)	81.3%	76.1%	78.6%	91.6%	**85.7%**	**88.6%**
BraiNER-W (features from WhiteText)	83.6% (3.3)	76.4% (4.6)	79.8% (3.9)	87.1% (3.6)	77.8% (7.4)	82.1% (5.8)
BraiNER (with additional features)	**84.6%** (1.3)	**78.8%** (1.2)	**81.6%** (0.9)	88.4% (1.0)	81.0% (1.8)	84.6% (1.3)

For machine learning-based NERs [French *et al.* (2009) and BraiNER], average values over 8-fold cross validation with splits at document level and 5 repetitions, including standard deviation in parenthesis where appropriate.

For machine learning NERs, we first reproduce the results from French *et al.* (2009), using the same model and features (github.com/leonfrench/public/). This model is denoted BraiNER-W and its performance is slightly higher than the results reported by French *et al.* (2009) for exact comparison (83.6% precision against 81.3% and 76.4% recall against 76.1%). This can be explained by the differences in pre-processing (tokenization, part-of-speech, abbreviation expansion). For lenient comparison, results from BraiNER-F are slightly worse, probably because we use a stricter lenient comparison criterion. Finally, we evaluate BraiNER that includes additional model features. Performance is slightly higher than BraiNER-W (e.g. *F* score 81.6% against 79.8% in strict comparison and 84.6% against 82.1% in lenient comparison). However, differences are not statistically significant. Nevertheless, qualitatively we found that the performance of BraiNER is higher when analyzing full-text articles.

Compared with lexical-based NERs, both machine learning-based NERs score slightly higher on precision, but have a much higher recall (more than twice as much). However, the low recall of lexical-based NERs is still acceptable for our purpose, since we apply theses NERs on very large corpora and focus on precision.

### 3.2 Connectivity Extractors Evaluation

The connectivity extractors are evaluated on the *WhiteText connectivity* corpus from French *et al.* (2012), that goes beyond the original *WhiteText* corpus and contains 3097 manually annotated connectivity relations across 989 abstracts and 4338 sentences from the *Journal of Comparative Neurology*. Inter-annotator agreement reaches a precision and recall of 93.9% and 91.9%, respectively (partially matching spans, two curators). In this evaluation, the locations of the brain region entities in the text are provided, so we are only concerned with the evaluation of the extractors.

Table 4 lists the evaluation results. The baseline connectivity extractor returns all permutations of two brain regions within a sentence and has a perfect recall of 100% but a very low precision of 9%. Note that French *et al.* (2012) estimated that over a forth of all connectivity relations are formed with regions spanning different sentences. Extracting connections that span sentences was not considered and the evaluation is performed without accounting for the relations spanning sentences. Subsequently, four filters are applied and evaluated. The first two (filter if sentence is longer than 500 characters or contains more than 7 brain regions) do not significantly improve precision on the evaluation corpus, but they proved very effective when dealing with full-text articles. The next filter requires certain trigger words (like *project*) to be present in the sentence and improves the precision to 15%. The last filter (keeping only nearest neighbors co-occurrences) improves the baseline precision (9%) 3-fold to 28%. When combining all filters (FILTERS), almost half of the extracted connections are correct (45% precision). However, only 31% of the connections are recalled.

**Table 4.** Evaluation of extraction models against the WhiteText corpus

Extractor	Prec.	Recall	*F* score
All co-occurrences (all permutations)	9%	**100%**	16%
Filter sentence > 500 characters	10%	93%	18%
Filter sentence with > 7 brain regions	11%	80%	19%
Keep if contain trigger words	15%	53%	23%
Keep nearest neighbor co-occurrence	28%	51%	36%
All filters (FILTERS)	45%	31%	37%
Shallow linguistic kernel (KERNEL)	60%	68%	**64%**
Ruta rules (RULES)	**72%**	12%	21%
FILTERS and KERNEL	66%	**19%**	**29%**
FILTERS and RULES	80%	7%	13%
KERNEL and RULES	81%	10%	18%
FILTERS and KERNEL and RULES	**82%**	7%	12%
(FILTERS or KERNEL) and RULES	80%	11%	19%

For the machine learning model (KERNEL), 10-fold cross-validation with splits at document level is performed, resulting in a precision of 60%. Recall (68%) is significantly higher than with FILTERS. Finally, RULES (manually created rules) yields the highest precision, at the cost of a very low recall. Still, this performance is quite remarkable, considering its simplicity (only nine rules).

Ensemble of extractors is also considered to improve precision. For example, the connections returned by all three extractors have a highest precision of 82% at only 7% recall. For connections returned by FILTERS or KERNEL, together with RULES, the performance is 80% precision at 11% recall.

### 3.3 Database

The models presented in this article are applied to two large corpora of biomedical literature. The resulting brain connectivity statements are stored in a database and an interface is created to navigate and make the results accessible to neuroscientists (see Fig. 1).

The two datasets consist of all PubMed article containing an abstract (13.2 million in total) and 630 216 neuroscientific full-text articles (Table 5). PubMed abstracts have the advantage to be available in large quantities and to capture the essential semantics of an article. On the other hand, full-text articles represent a very important source of connectivity information, as they potentially contain more connectivity statements. We found on average 6.4 times more connections in full-text articles than in abstracts. The full-text corpus used is focused on neuroscience and was created by aggregating articles from the personal libraries of all researchers in our research institute. This process was facilitated by the massive collaborative use of Zotero (www.zotero.org). In addition, full-text articles containing mentions of ABA brain regions were collected from the PubMed Central Open Access Subset and from open access journals related to neuroscience.

**Table 5.** Statistics of the corpora used, extracted brain regions and connections using all three extractors (FILTERS or KERNEL or RULES)

Corpus	Corpus statistics		Brain regions			Connectivity statements		
	Documents	Words	ABA	BAMS	BraiNER	ABA	BAMS	BraiNER
All PubMed abstracts	13 293 649	2.1 × 10^9^	1 705 549	1 918 561	1 992 747	41 965	50 331	188 994
Full-text neuroscience articles	630 216	6.1 × 10^9^	2 327 586	2 514 523	2 751 952	62 095	72 602	279 100

The number of documents and words refers to non-empty documents after pre-processing. Two generic terms from BAMS (brain and nerves) are omitted.

**Fig. 2.**
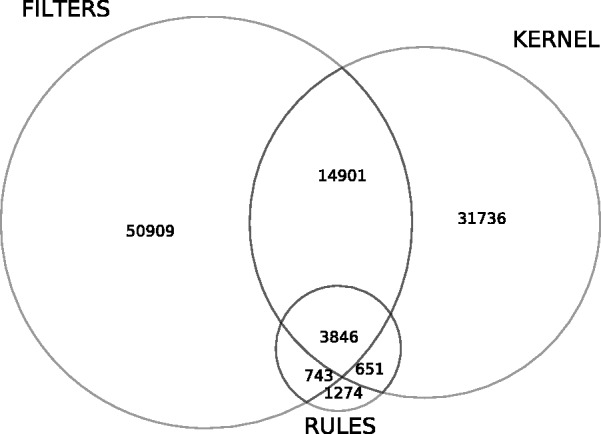
Number of extracted connections for the three extractors, on PubMed and full-text corpora using the ABA-SYN NER

Connections are extracted using Bluima, an integrated suite of software components for natural language processing (NLP) of neuroscientific literature (Richardet *et al.*, 2013). The processing is distributed on a cluster and the extraction results are aggregated in a database. The resulting database contains several million brain region mentions (Table 5). In the PubMed abstracts, 42, 50 and 189 000 connection pairs are extracted for ABA, BAMS and BraiNER, respectively. For the full-text neuroscience corpus, 62, 72 and 279 000 connection pairs are extracted for ABA, BAMS and BraiNER, respectively. Figure 2 highlights the overlap of the results from all three extractors. For example, 31 736 connections are extracted uniquely by KERNEL, whereas all three extractors return 3846 connections. Thus, each extractor contributes to extracting a different set of brain region connections, with a different performance. This will turn out to be useful to display connectivity data: the connections that are returned by all three extractors have a higher estimated precision and ought to be displayed at the top of the list of proposed results.

The database is accessible through a web service, with a simple web front end. It allows neuroscientists to search for a given region and display all other connected regions. It also allows to provide a feedback on the results for future model improvements. Normalization and standardization of brain region entities identified by BraiNER can be manually performed by the user (no morpho-syntactic transformation).

### 3.4 Database evaluation against AMBCA

Results extracted from the literature (LIT) are evaluated against connectivity data from the AMBCA. The AMBCA validation corpus consists of the normalized connectivity data from 469 *in* *vivo* experiments [See Oh *et al.* (2014), Supplementary Table S3 for the underlying data.]. Regions were filtered by two criteria (bigger than 50 voxels and containing enough data for the signal to be well linearly separable), resulting in 213 selected regions (out of a total of 1204 regions in the complete ontology). Thus, AMBCA consists of a square matrix of 213 brain regions, whose values represent normalized ipsilateral connection strengths. In total, 16 954 brain region pairs are reported as connected (37%) and 28 415 as not connected.

The evaluation of LIT against AMBCA proved to be quite complex. First, it is not possible to determine which articles are missing in LIT (i.e. articles that should have been retrieved by LIT but were missed). Therefore, it is not possible to correctly evaluate the *recall* of LIT. Second, AMBCA contains 213 regions, whereas LIT contains 451 regions, thus 238 regions from LIT cannot be evaluated and were removed from the evaluation. Third, many ABA brain regions never occur in the literature (mainly because they are very specific, like ‘Anterior cingulate area, dorsal part, layer 2/3’). In fact, half of the ABA regions (603 out of 1204) are never found in the literature by the ABA lexical NERs. Forth, AMBCA uses one single and systematic experimental method, whereas many different methods and experimental settings are reported in scientific reports from AMBCA, making the comparison problematic. Fifth, it is important to highlight that the *frequency* of a brain region connection reported in scientific articles does not necessarily reflect the *physiological intensity* of a connection; the former reflecting the *popularity* of a region.

**Fig. 3.**
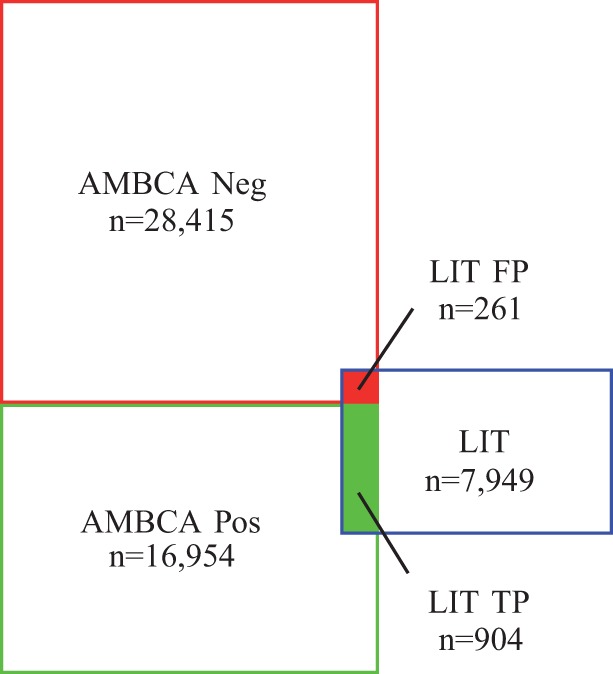
Evaluation against AMBCA. AMBCA contains 16 954 distinct connected brain region pairs (AMBCA Pos) and 28 415 unconnected pairs (AMBCA Neg). Connectivity data extracted from the literature contain 7949 distinct connected brain region pairs (LIT), of which 904 are connected in AMBCA (LIT TP) and 261 are not connected in AMBCA (LIT TN)

Despite all these limitations, the evaluation is highly relevant, as it allows to compare our models with experimental data. Figure 3 illustrates the evaluation results. 904 brain region pairs are correctly predicted (present in LIT and connected in AMBCA) and 261 brain region pairs are incorrectly predicted (present in LIT but not connected according to AMBCA), resulting in a 78% precision, which is an impressively good result regarding the five previously mentioned limitations of this evaluation. In comparison, the precision of co-mentioned brain region mentions (two brain regions within the same sentence, without any filtering) is 67%. By thresholding co-mentions to those predicted at least four times, precision reaches 72%, suggesting that frequent co-mentions can successfully predict connectivity.

The 6784 brain region pairs present in LIT but not in AMBCA (represented as the blue square with white background in Fig. 3) are valuable connections that might complement experimental datasets like AMBCA. However, it is impossible to quantitatively evaluate these brain regions because of the lack of objective reference. Furthermore, when using another NER like BrainNER, even more brain regions (not present in AMBCA) would be retrieved, resulting in an even larger size of LIT.

**Fig. 4.**
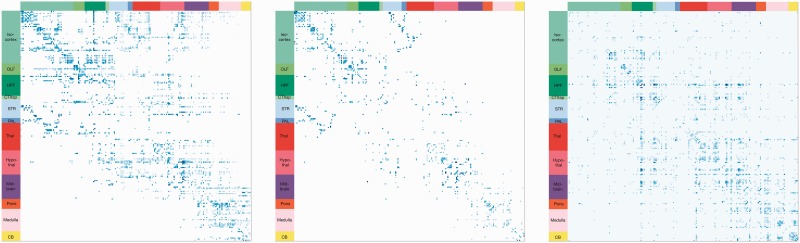
Comparison of the inter-region connectivity matrices, renormalized between 0 (white) and 1 (blue). Rows and columns correspond to ABA brain regions. *Left:* connection matrix from AMBCA (ipsilateral), using ABA’s inter-region connectivity model, with values representing a combination of connection strength and statistical confidence [see Fig. 4a of Oh *et al.* (2014)]. *Middle:* same matrix from AMBCA, but symmetrized (connection directionality is ignored, since the NLP models do not extract directionality). *Right:* connection matrix from the results extracted from the literature (LIT) with values representing the number of extracted connectivity statements, weighted by the estimated precision of each connectivity extractor

Figure 4 shows the *in* *vivo* connectivity matrix from AMBCA (left), the symmetrized matrix from AMBCA (middle, required, to compare against the NLP models that do not extract directionality) and the *in* *litero* connectivity matrix extracted from the literature (LIT, right). The LIT matrix is much sparser than AMBCA, as was previously noted. However, both matrices exhibit a similar structure. To evaluate this similarity, the precision between LIT and AMBCA (symmetrized) matrices are compared against 1000 random matrices created by shuffling the brain region names in the same way for rows and columns. That ensures symmetry with the same node degree distribution and density. LIT is significantly closer to AMBCA than the random matrices (*P* < 0.01).

No significant difference in precision can be observed between the connections originating from abstracts and the ones from full-text articles. Similarly, no significant difference in distance can be observed between abstracts and full-text articles. We also evaluate the depth of the extracted connections, measured as the mean number of parents (higher structures) in the ABA ontology. Connections from AMBCA have a mean depth of 6.21, whereas connections extracted from the literature have a depth of 5.08.

## 4 Discussion

We demonstrate that an exploitable brain region connectivity database can be extracted from a very large amount of scientific articles. Our models extract large amounts of connectivity data from unstructured text and compare favorably against *in* *vivo* connectivity data. They provide a helpful tool for neuroscientists to facilitate the search and aggregation of brain connectivity data.

Our work builds on top of French *et al.* (2009, 2012) and French and Pavlidis (2012) and extends it in several aspects: Our connectivity extraction model uses a combination of three different extractors, including a novel rule-based extractor that achieves state-of-the-art precision. Models were applied to a comprehensive corpus of over 8 billion words, consisting of all available PubMed abstracts and a very large number of full-text articles related to neuroscience. New model features and extraction filters were added to improve robustness on full-text extraction. Connectivity results are presented to neuroscientist in an interface to rapidly search and evaluate connectivity results.

We highlight the fact that the presented models are not meant to replace manual and individual evaluation of the connectivity between two brain regions. The objective is to speed-up this evaluation and complement *in* *vivo* or manually curated connectivity data. We assume that the extracted connectivity data will be reviewed and validated before being included in further analysis or models. Manual review is also mandatory since connection extractors have a very limited capacity to differentiate between hypothesized or contradictory connections, connections referred from another article or connections supported by experimental data. Therefore, the efficient representation of connectivity data is important, so that domain experts can rapidly evaluate it.

A drawback of manual search (as it is most commonly performed for literature search) is the inability to provide *feedback* on search results. More that 3 million manual searches are performed *daily* on the PubMed web site (www.nlm.nih.gov/services/pubmed_searches.html). Yet, a manual search performed by a researcher will neither improve future searches nor contribute to the building and curation of a structured knowledge base. In contrast, our database interface allows researcher to rate search results (collaborative filtering). Once enough feedback data are collected, the models can be retrained to achieve even higher performance.

This study highlights the differences in complexity and performance between machine learning and rule-based approaches. The former delivers superior performance but requires a significantly more complex setup, in particular in terms of knowledge required (model and feature selection) and time for corpus annotation and model training. On the other hand, rule-based approaches are much simpler and require less time to develop. They are also less tightly bound to the domain they are applied to. For example, the *FILTERS* extractor (Section 3.2) could be applied to relationship extraction between other entities (like neurons or proteins) without significant modification. However, the performance of rule-based approaches is significantly lower, especially in terms of recall.

In the future, we plan to extend the developed rule-based extractors with a large-scale data-driven strategy. We also plan to apply the presented models to support the selection of relevant seed regions when performing magnetic resonance imaging experiments.

## Supplementary Material

Supplementary Data
