# Supplementary material for: Large-scale extraction of brain connectivity from the neuroscientific literature
Source: Bioinformatics. 2015 Jan 20;31(10):1640–7. doi: 10.1093/bioinformatics/btv025 (PMC4426844; doi:10.1093/bioinformatics/btv025)
Supplement: Supplementary Data [file supp_31_10_1640__index.html]

Large-scale extraction of brain connectivity from the neuroscientific literature — Large-scale extraction of brain connectivity from the neuroscientific literature — Large-scale extraction of brain connectivity from the neuroscientific literature — Supplementary Data 

# Large-scale extraction of brain connectivity from the neuroscientific literature

## Supplementary Data

files

**Files in this Data Supplement:**

- Supplementary Data - zip file
