# Supplementary figures and images for: Large-scale extraction of brain connectivity from the neuroscientific literature
Source: Bioinformatics. 2015 Jan 20;31(10):1640–7. doi: 10.1093/bioinformatics/btv025 (PMC4426844; doi:10.1093/bioinformatics/btv025)

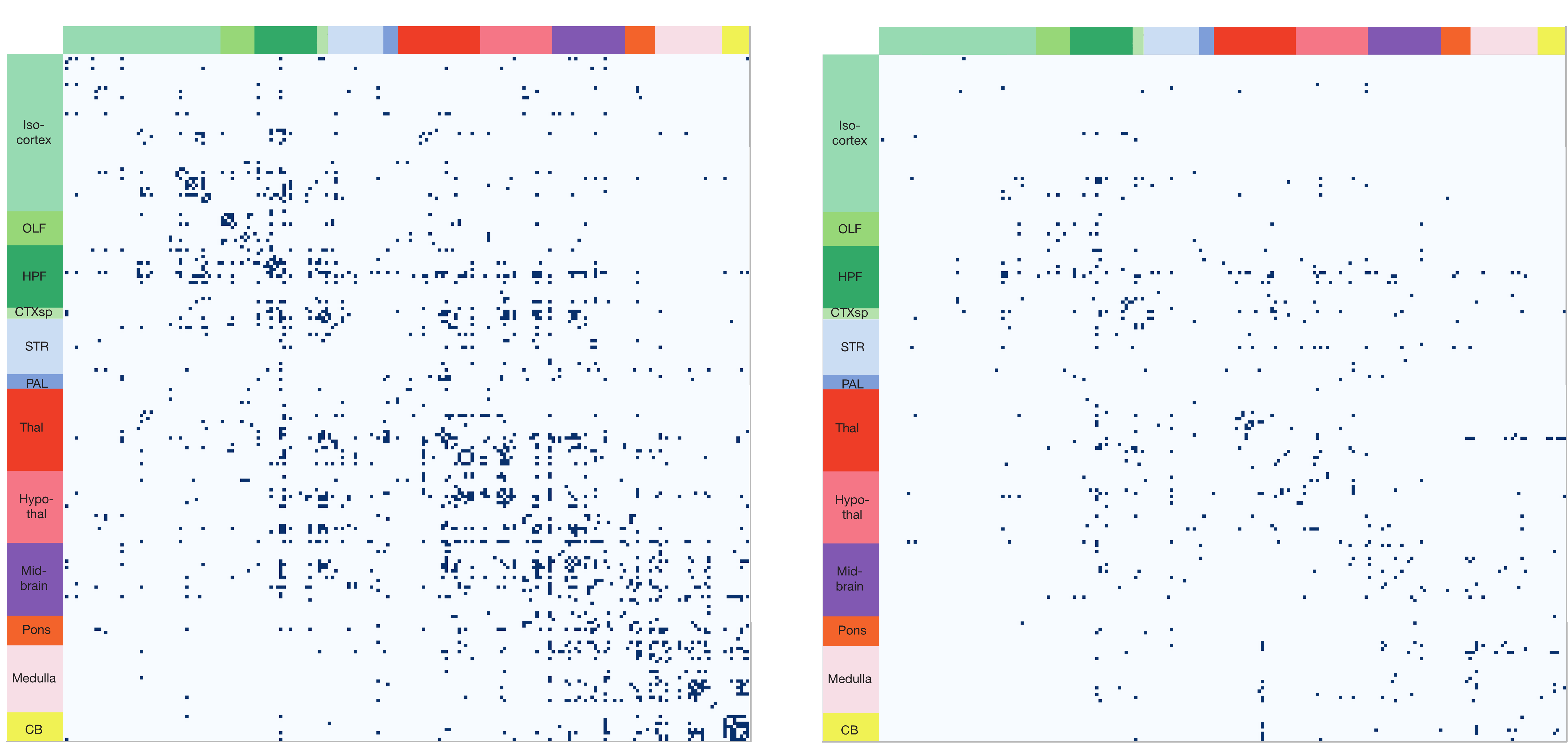

Supplement: Supplementary Data [file supp_btv025_supplementary.zip › supplementary/matrices_tp-fp.png]

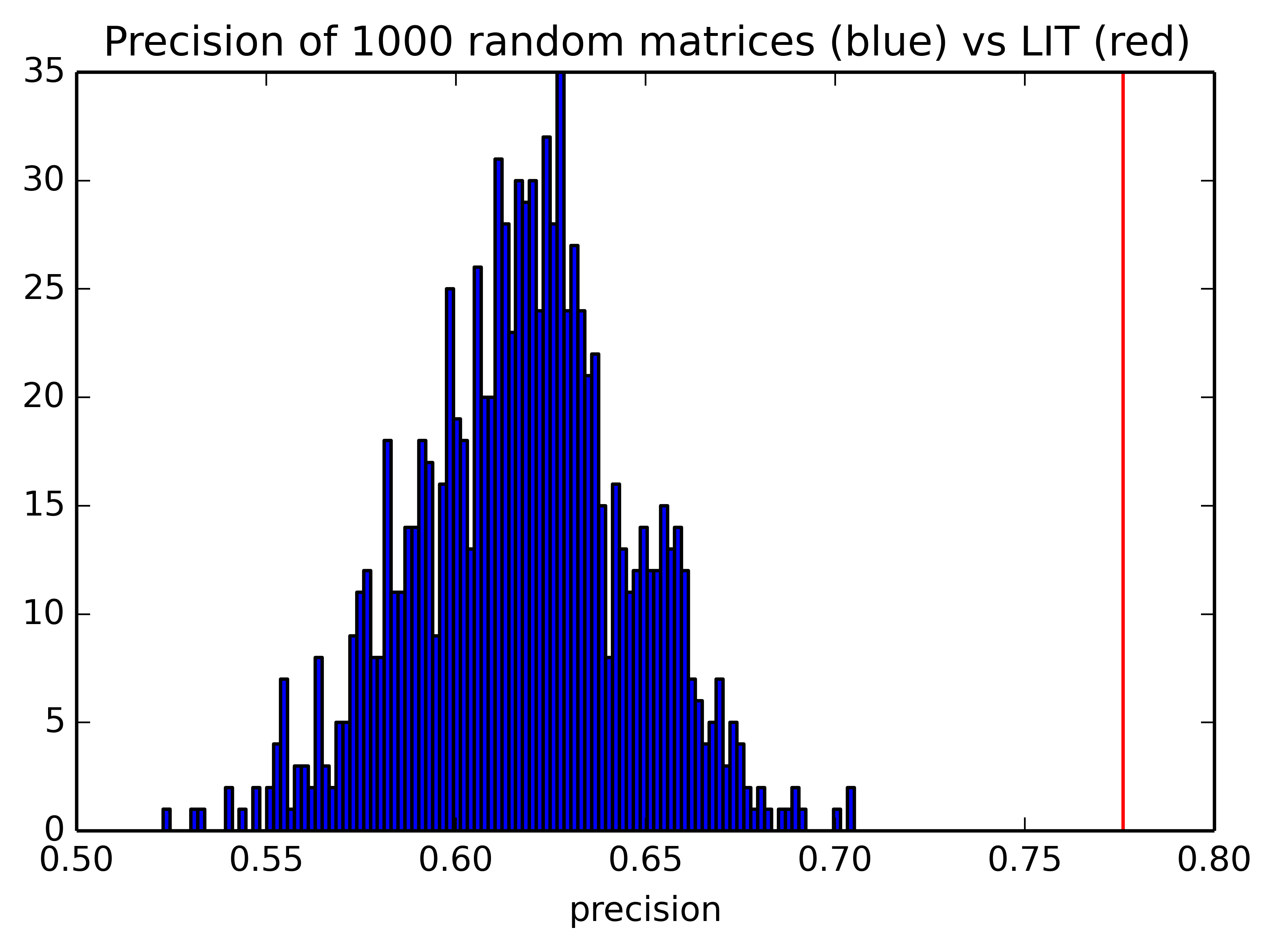

Supplement: Supplementary Data [file supp_btv025_supplementary.zip › supplementary/precision_against_random.png]
